# A Linear Dose-Response Relationship between Fasting Plasma Glucose and Colorectal Cancer Risk: Systematic Review and Meta-analysis

**DOI:** 10.1038/srep17591

**Published:** 2015-12-01

**Authors:** Jianguo Shi, Lijuan Xiong, Jiaoyuan Li, Heng Cao, Wen Jiang, Bo Liu, Xueqin Chen, Cheng Liu, Ke Liu, Guobin Wang, Kailin Cai

**Affiliations:** 1 Department of Gastrointestinal Surgery, Union Hospital, Tongji Medical College, Huazhong University of Science and Technology, Wuhan 430000, China; 2 State Key Laboratory of Environment Health (Incubation, Tongji Medical College, Huazhong University of Science and Technology, Wuhan 430000, China; 3 Ministry of Education) Key Laboratory of Environment & Health, Tongji Medical College, Huazhong University of Science and Technology, Wuhan 430000, China; 4 Ministry of Environmental Protection Key Laboratory of Environment and Health (Wuhan), Tongji Medical College, Huazhong University of Science and Technology, Wuhan 430000, China; 5 Department of Epidemiology and Biostatistics, School of Public Health, Tongji Medical College, Huazhong University of Science and Technology, Wuhan 430000, China; 6 Department of Infectious Disease, Union Hospital, Tongji Medical College, Huazhong University of Science and Technology, Wuhan 430000, China

## Abstract

For many years, the question of whether hyperglycaemia, a manifestation of prediabetes, diabetes mellitus and metabolic syndrome, is a risk factor for colorectal cancer has been intensely studied. In fact, even after the conclusion of several prospective studies, the topic is still controversial. We conducted a systematic review and meta-analysis to investigate the dose-response relationship between blood glucose concentration and the incidence of colorectal cancer. A linear (*P* = 0.303 for non-linearity) dose-response relationship was observed between fasting plasma glucose (FPG) and colorectal cancer risk without significant heterogeneity. The relative risk (RR) for colorectal cancer per 20 mg/dL increase in FPG was 1.015 (95% CI: 1.012–1.019, *P* = 0.000). In subgroup analyses, the pooled RRs for colon cancer (CC) and rectal cancer (RC) studies were 1.035 (95% CI 1.008–1.062, *P* = 0.011) and 1.031 (95% CI: 0.189–5.628, *P* = 0.972), respectively; in the analysis comparing men and women, the pooled RRs were 1.016 (95% CI: 1.012–1.020, *P* = 0.000) and 1.011 (95% CI: 0.995–1.027, *P* = 0.164), respectively. Sensitivity analyses using two methods showed similar results. In conclusion, there is a significant linear dose-response relationship between FPG and the incidence risk of colorectal cancer. For people with diabetes or prediabetes, controlling blood glucose might be useful to prevent colorectal cancer.

## Introduction

Colorectal cancer was the third most common cancer diagnosed in 2012 and the fourth leading cause of cancer death worldwide, according to GLOBOCAN 2012. Some factors, such as ageing, genetic factors, external carcinogens, unhealthy diet, and physical inactivity, have been reported as risk factors for colorectal cancer^1^,^2^,^3^,^4^,^5^. Many epidemiological studies have also shown that prediabetes, diabetes mellitus (mainly type 2, T2DM) and metabolic syndrome might raise the incidence and mortality rates of colorectal cancer^6^,^7^,^8^,^9^,^10^. The global prevalence of diabetes among adults is 8.3% (382 million)^11^, and metabolic syndrome prevalence ranges from 10% to 40%^12^,^13^,^14^. “Prediabetes” is a condition with impaired fasting glucose (IFG) and/or impaired glucose tolerance (IGT) that tends to meet the diagnosis criteria for diabetes^15^. The data on a large population of prediabetics were published in 2013: 316 million (6.9%) adults 20–79 years old had IGT^16^. Hyperglycaemia resulting from a Western diet and insulin resistance was a common clinical presentation and an important pathophysiological factor among the three conditions^17^,^18^,^19^,^20^,^21^. Therefore, whether hyperglycaemia influences the incidence of colorectal cancer is critically important for cancer prevention.

Several prospective studies have reported different blood glucose levels and the corresponding incidences or relative risks (RRs) of colorectal cancer. However, the exact relationships between blood glucose concentration and colorectal cancer risk in each study were controversial^22^,^23^,^24^,^25^,^26^,^27^ and have not been systematically reviewed. Additionally, some studies have indicated that the diabetes mellitus state with hyperglycaemia or even without hyperglycaemia due to use of antihyperglycemic medications influenced the risk of cancers such as colorectal cancer^28^,^29^. Therefore, diabetes mellitus was considered a confounding factor when examining the influence of blood glucose concentration on colorectal cancer risk. However, only one of these prospective studies excluded the diabetes mellitus population at the recruitment stage^26^.

In this study, we conducted a systematic review with dose-response meta-analysis to provide more reliable and precise estimates of the relationship between blood glucose concentration and colorectal cancer risk^30^,^31^.

## Methods

### Search strategy and exclusion/inclusion criteria

We conducted a meta-analysis on the basis of the proposed reporting checklist from the MOOSE Meta-analysis of Observational Studies in Epidemiology (MOOSE) group^32^. The PubMed and EMBASE databases were independently searched before 10 February 2015 without language or time restrictions but with one limitation: only publications examining human data were considered. After reading the titles and abstracts of all of the studies, we first excluded retrospective and cross-sectional studies. Full manuscripts of prospective studies and systematic reviews were obtained and scrutinized to assess the association between blood glucose concentration and the incidence of colorectal cancer. Useful references in full-text articles that were not captured in the first step of the electronic search were found manually. We also screened studies by researchers who cited eligible studies. The detailed search strategy and inclusion/exclusion criteria are included in the Appendix. We resolved disagreements through joint discussions among all of the authors in the meta-analysis.

### Data extraction and study quality assessment

**Figure 1:**
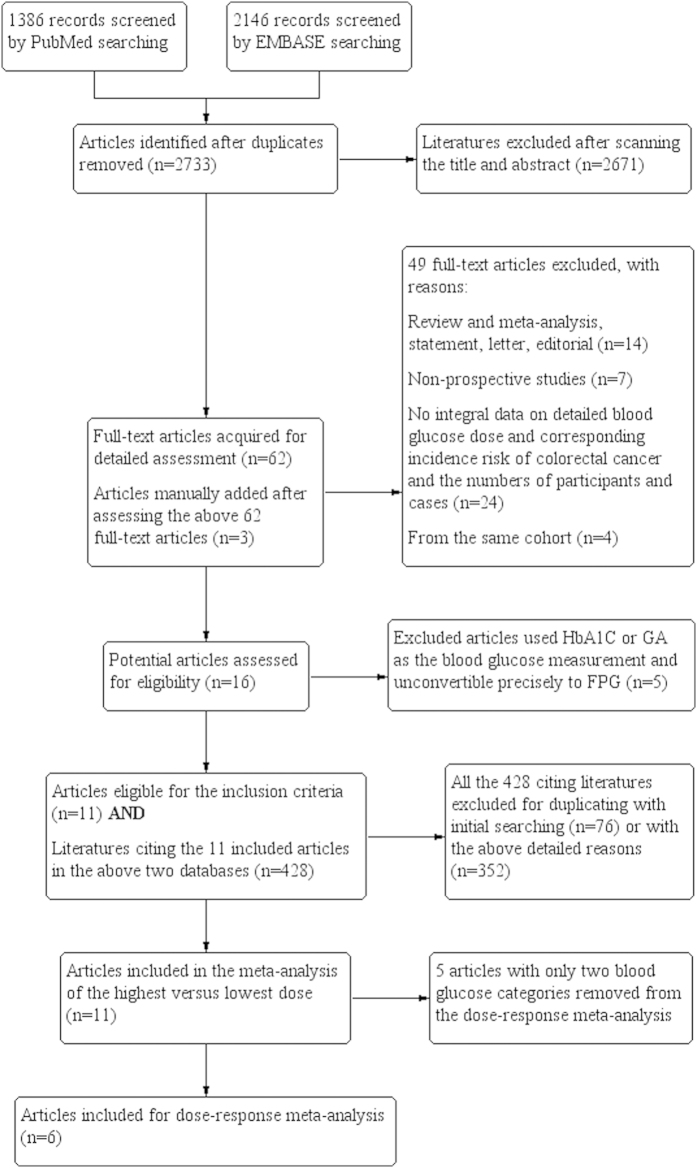
Flow diagram of literature screening.

The following data were independently extracted from eligible studies: first author, publication year, region of the recruited population, cancer type, study design, recruitment baseline, follow-up time, whether the diabetes mellitus population was excluded, age at recruitment, gender (men%), values of fasting plasma glucose (FPG) or HbA1C, fasting state, the size of the observational population (N) and the number of subsequent colorectal cancer cases, types and values of relative risk and 95% confidence intervals, and adjusted confounders. If a study reported results for colon cancer and rectal cancer, we separated the article into two independent studies by cancer site. If an article reported results for different genders, we considered them separately. In terms of FPG, fasting is currently defined as having no caloric intake for at least 8 h^15^. However, we recorded the fasting state in light of the definition in the original studies given the time and regional differences among eligible studies. If the included articles reported on several models, such as a crude model and an adjusted model, we adopted the model with much confounders adjusted. We evaluated the quality of each study with the Newcastle-Ottawa Quality Assessment Scale for cohort studies (see Fig. 1 in the Appendix)^33^.

### Statistical analysis

For the dose-response meta-analysis, we used the generalized least squares trend (GLST) model proposed by Greenland and Longnecker^34^,^35^ to estimate the trend in the effect. Based on the construction of an approximate variance-covariance matrix for the log relative risk, this approach could be employed to obtain a corrected linear association using general least squares. A cubic spline model with 3 knots at the 25%, 50% and 75% percentiles of the distribution was established to explore the potential non-linear relationship between glucose concentration and the relative risk for colorectal cancer, and a *P* value for non-linearity was calculated by testing the null hypothesis that the coefficient of the second spline was equal to zero^36^. For each study, the median or midpoint of the upper and lower boundaries was assigned as the mean glucose concentration in each category. If the lower boundary of the lowest category was not available, it was then defined as 70 mg/dL, which is the lower limit of normal blood glucose concentration^37^. When the upper boundary of the highest category was not provided, it was calculated as the lower bound plus 1.5 times the width of the neighboring category. We unified FPG as the exposure indicator because it is a common diagnostic criterion for prediabetes and T2DM and provides one common laboratory value to describe the metabolic syndrome. All of the studies that reported only HbA1C^38^,^39^,^40^,^41^ or glycoalbumin (GA)^42^ for blood glucose concentration were discarded, as we could not accurately convert them into FPG data (see the Appendix for data synthesis and analysis).

The heterogeneity across studies was assessed by Cochran’s Q test and *I*^*2*^ statistic. The criterion for identifying heterogeneity was a *P* value less than 0.05 for the Q test or an *I*^*2*^ value greater than 50%. When significant heterogeneity was detected, data from the included studies were combined in a random-effects model; otherwise, the fixed-effects model was employed. We conducted subgroup analysis to search for the source of heterogeneity, and the subgroups were pre-specified mainly according to cancer type, gender, region and follow-up time. Sensitivity analysis was also performed to evaluate the stability of associations. Moreover, we completed a meta-analysis of the studies with two-category variables (highest compared to lowest blood glucose level). Because the comparison groups were quite different, it would not make sense to pool together studies reporting several categories (FPG category ≥3) and only a dichotomous variable (FPG category = 2) for blood glucose. Thus, we divided the meta-analysis of two-category variables into two parts according to the total number of original FPG categories. Publication bias was examined with Begg’s and Egger’s regression tests.

All of the analyses were performed with Stata 10.0 software. All of the *P* values were two-sided, and *P* < 0.05 was considered statistically significant.

## Results

A total of 2,733 articles were identified after duplicates were removed in the initial search (Fig. 1). Moreover, we manually added 3 potential manuscripts after a detailed evaluation of full-text articles. Additionally, 428 eligible citing articles were screened. The detailed justifications for exclusion are described in the Appendix. In the end, our study included 11 articles reporting results for 4,462,151 participants (67,190 colorectal cancer cases)^22^,^23^,^24^,^25^,^26^,^27^,^43^,^44^,^45^,^46^,^47^, including 6 articles that were included in the dose-response meta-analysis, comprising 2,969,306 participants (62,814 colorectal cancer cases)^22^,^23^,^24^,^25^,^26^,^27^. Each of the eligible articles was awarded at least eight stars according the Newcastle-Ottawa Quality Assessment Scale for cohort studies. Therefore, all of the articles were regarded as high quality (see Fig. 1 in the Appendix).

We divided the 11 articles into 21 studies because 8 articles were straight stratified according to cancer type and/or gender^23^,^24^,^25^,^27^,^43^,^45^,^46^,^47^. Table 1 shows the essential characteristics of the included studies. The mean follow-up time of all of the cohorts ranged from 3.7 to 12.8 years. All of the studies used the FPG value as a measurement of glucose level. Fifteen studies measured the blood glucose concentration of participants only in the fasting state, whereas the other 6 studies contained fasting or non-fasting data. The detailed relative risks of colorectal cancer for different fasting plasma glucose doses are presented in Table 1 of the Appendix.

### Meta-analysis

**Figure 2:**
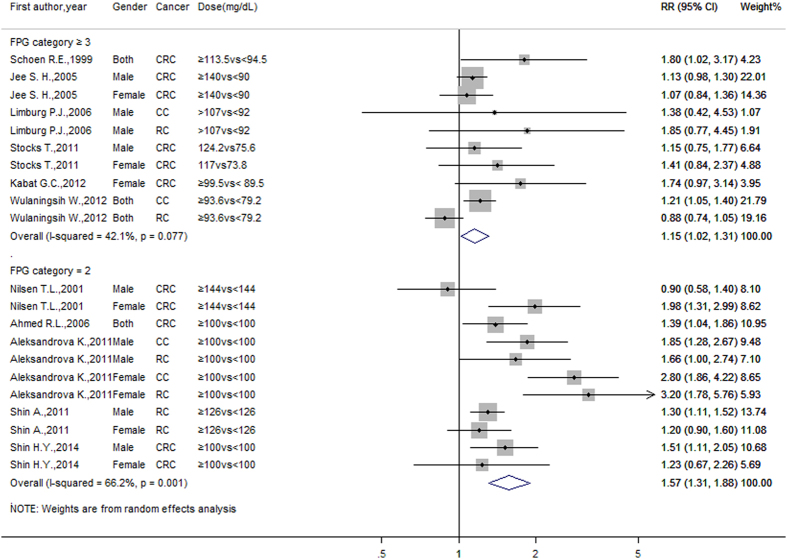
Summary risk ratios for colorectal cancer, the highest compared to lowest FPG category. Considering that the doses of the comparison groups were quite different, we divided the meta-analysis of two-category variables into two parts according to the total number of original FPG categories (FPG category ≥3 and FPG category = 2). There was slight or significant heterogeneity among these studies. The results should be interpreted critically.

We found a significant association between fasting plasma glucose and the incidence of colorectal cancer. For dose-response analysis, there was no evidence of departure from linearity among the data from the included studies (*P* = 0.303 for a non-linear trend). The summary RR for each 20 mg/dL increase in blood glucose concentration was 1.015 (95% CI: 1.012–1.019, *P* = 0.000), with little heterogeneity among studies (*I*^*2*^ = 11%, *P* = 0.295). For the meta-analysis of two-category variables, the combined RR for the highest glucose category of 10 studies (FPG category ≥3) was 1.152 (95% CI: 1.016–1.306, *P* = 0.027) compared to the lowest category; for the other 11 studies with FPG category = 2, the combined RR was 1.569 (95% CI: 1.307–1.885, *P* = 0.000). These results were consistent with the findings of the dose-response meta-analysis. However, slight (*I*^*2*^ = 42.1%, *P* = 0.077) and significant (*I*^*2*^ = 66.2%, *P* = 0.001) levels of heterogeneity were detected. The study-specific RRs per 20 mg/dL increase in blood glucose concentration are presented in Table 2, and the combined RRs for the highest compared to the lowest FPG are shown in Fig. 2 below.

### Subgroup analysis

Although no significant heterogeneity was detected across the 10 studies included in our dose-response analysis, subgroup analyses were further conducted according to cancer type, region, duration of follow-up, gender, fasting status and risk type. All of the related results are summarized in Table 3 below. For combined CRC and CC studies, positive dose-response relationships between FPG and cancer risk were reported (the pooled RRs were 1.016 (95% CI: 1.012–1.019, *P* = 0.000) and 1.035 (95% CI: 1.008–1.062, *P* = 0.011), respectively). However, no significant relationship was found after pooling RC studies. When stratified by gender, the RR estimates were 1.016 (95% CI: 1.012–1.020, *P* = 0.000) for males and 1.011 (95% CI: 0.995–1.027, *P* = 0.164) for females. Additionally, significant associations between blood glucose and CRC risk were detected for studies restricted to fasting status, a follow-up period of more than 10 years or a location in North America or Asia.

### Sensitivity analysis and publication bias

**Figure 3:**
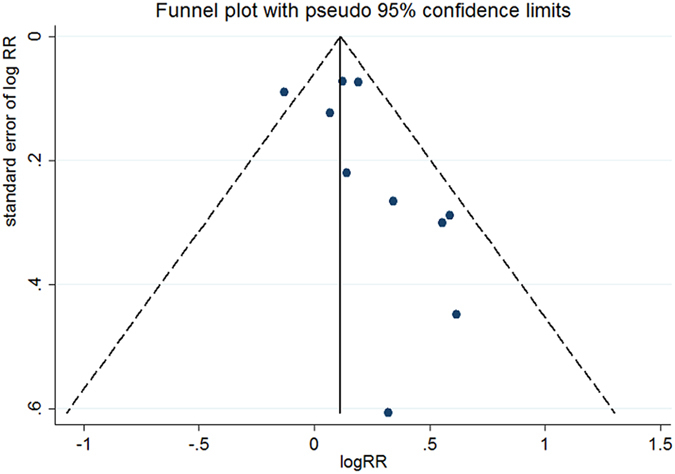
Funnel plot of the highest compared to lowest FPG categories from only 10 studies included in the dose-response meta-analysis. The funnel plot was based on 4 small studies (fewer than 100 cancer cases for each exposure dose) and 6 large studies. Among the 10 published studies, the results of 2 studies were statistically significant (*P* < 0.05), and the results of 8 studies were not significant (*P* > 0.05). For the 2 studies with significant results, one study was small, whereas the other came from the group of 6 large studies. No significant publication bias was detected (*P* = 0.125 for Egger’s test, *P* = 0.283 for Begg’s test). This funnel plot shows asymmetry, which might be related to reasons other than publication bias.

For the dose-response meta-analysis, no individual study could alter the linear trend when it was removed from the meta-analysis. The pooled RRs ranged from 1.013 (95% CI: 1.002–1.025, *P* = 0.021) to 1.016 (95% CI: 1.012–1.020, *P* = 0.000), and the relevant levels of heterogeneity remained insignificant (see Table 2 of the Appendix). After we excluded the exposure categories with fasting blood glucose ≥126 mg/dL (the FPG diagnostic cutoff point for diabetes mellitus) from the 10 included studies, we also observed a linear dose-response relationship (*P* = 0.092 for a non-linear trend) between FPG and the incidence of colorectal cancer (the summarized RR was 1.015 (95% CI: 1.011–1.019, *P* = 0.000) with no significant heterogeneity (*I*^2^ = 19, *P* = 0.213)). These findings confirmed the stability of our results. Neither Egger’s regression test nor Begg’s test detected significant evidence of publication bias (*P* = 0.125 for Egger’s test, *P* = 0.283 for Begg’s test) (Fig. 3).

## Discussion

We performed a meta-analysis of high compared to low levels and dose-response relationships between FPG and the incidence of colorectal cancer, and a linear dose-response relationship was identified after pooling six articles including 9,618 colorectal cancer cases. For the dose-response meta-analysis, an FPG increase of 20 mg/dL was associated with a 1.5% increase in the risk of colorectal cancer. Sensitivity analyses showed similar results. No significant heterogeneity or publication bias was observed across the 10 prospective studies. For the meta-analysis of high compared to low levels, higher blood glucose exposure indicated a higher risk of colorectal cancer than lower exposure. However, this result should be interpreted critically, considering that the FPG doses of comparison groups were quite different and that slight or significant heterogeneity was detected.

According to the RR or HR values for each dose category in each eligible study of the dose-response analysis, the association between fasting plasma glucose and the incidence risk of colorectal cancer was not significant in 5 studies^24^,^25^,^26^ and was mixed in another 5 studies^22^,^23^,^27^ (see Table 1 in the Appendix). Even in the study-specific dose-response meta-analysis using a 20 mg/dL incremental increase of FPG, the variation in the incidence of colorectal cancer was significant in 4 studies (*P* < 0.05) but not significant in 6 studies (*P* > 0.05) (Table 2). However, our meta-analysis showed no significant heterogeneity across the 10 prospective studies. Therefore, it was suitable to pool the 10 studies, and there were several potential explanations for these seemingly inconsistent results. First, the number of blood glucose categories and the corresponding size of the colorectal cancer cases and observational population were distinct in different studies. Hence, we should not directly compare the RR or HR values for different studies. Second, the number of colorectal cancer cases or the size of the observational population was small in individual studies; thus, the RRs or HRs had low power, and their 95% confidence intervals were wide. The results from individual studies might not accurately reflect the actual situation of the global population. Additionally, these studies differed with respect to region, age at recruitment, cancer type and gender.

Our dose-response meta-analysis showed that the incidence risk of colorectal cancer did increase with the elevation of blood glucose concentration, implying that blood glucose was a dose-dependent risk factor for the incidence of colorectal cancer, which is consistent with previous studies of the association between prediabetes^6^ and diabetes mellitus^48^,^49^ and colorectal cancer risk. Additionally, the value of the pooled RR was almost unchanged when we excluded the exposure categories with fasting blood glucose ≥126 mg/dL. This result confirmed that hyperglycaemia, instead of the diabetes mellitus state, influenced the incidence of colorectal cancer. Several possible mechanisms could explain the association between hyperglycaemia and increasing colorectal cancer incidence. High glucose concentration could induce DNA damage independent of insulin in human endothelial cells^50^, and glucose catabolism could trigger quiescence exit and therefore could be a critical factor for cell proliferation^51^. It was shown that hyperglycaemia could, independent of insulin, enhance the cancer-associated Wnt/β-catenin signalling pathway through glucose-dependent β-catenin nuclear retention in macrophage and enteroendocrine cell lines^52^,^53^. High glucose exposure can promote the migration and invasion of rat colorectal cancer cells and the STAT3-induced MMP-9 signalling pathway involved in that process^54^. Glucose deprivation might also influence carcinogenesis; for example, it reduces the stability and expression of the LGR5 protein on the cell surface, a crucial marker of intestinal stem cells that is required for enhanced Wnt signalling^55^.

For subgroup analyses of the dose-response meta-analysis, the incidence of colorectal cancer increased with rises in FPG concentration in CRC and CC studies, studies performed in North America and Asia, studies with male participants and with a follow-up time ≥10 years; however, the results were uncertain for RC studies, studies conducted in European contexts, and studies with female participants. These results were consistent with the findings of previous studies on diabetes mellitus or metabolic syndrome (including hyperglycaemia) and colorectal cancer risk and time-dependent analyses^6^,^7^,^56^,^57^,^58^. Variations in metabolic status in different segments of the intestines, hormone levels in different genders and diet habits in different areas might be responsible for the differences observed in the subgroup analysis.

Some strengths of this study should be acknowledged. We conducted a dose-response meta-analysis (quantitative review) of published prospective studies on blood glucose concentration and colorectal cancer risk and found a positive linear relationship between them, which is the first time to our knowledge. Although all of the eligible studies analysed the linear trend, the results varied, and most of them did not indicate a quantitative dose-response relationship. All of the eligible studies were high-quality prospective studies. Therefore, the possibility of reverse causality need not be considered. The total number of participants (n = 2,969,306) and colorectal cancer cases (n = 9,618) was sufficiently large, and these subjects were from different regions throughout the world (Europe, North America and Asia). The measure of blood glucose concentration was consistent (FPG) in the included studies. No individual study was able to alter the linear trend when we removed it from the dose-response meta-analysis. To avoid the interference of diabetes mellitus status, we performed another sensitivity analysis after excluding the exposure categories with fasting blood glucose ≥126 mg/dL, and the results were similar to our primary results. Thus, the pooled RRs of the 10 studies were stable and reliable.

Our study also had several limitations. Among the 10 studies included in our dose-response meta-analysis, the participants’ FPG was measured at baseline in only 5 studies^23^,^25^,^26^, and such studies therefore could not provide an accurate estimate of their FPG value over a relatively long follow-up period, especially for participants who were diagnosed with diabetes or participants who controlled their blood glucose deliberately after being recruited. Therefore, to increase the accuracy of the results, it is preferable to measure participants’ FPG repeatedly until the end of the follow-up period, as did the other 5 studies, and then calculate the mean FPG value for each participant with respect to exposure level. Site-specific risks for CC and RC were analysed in only 2 articles. Therefore, the results of the dose-response meta-analysis for CC or RC studies in subgroup analyses should be critically examined, especially for RC (Table 3). Although we excluded the exposure categories with fasting blood glucose ≥126 mg/dL to avoid the interference of diabetes mellitus to the greatest extent possible, information on the use of antihyperglycemic medications was not available in most of the eligible studies. Therefore, it was possible that individuals with diabetes mellitus taking antihyperglycemic medications might have been included in the sensitivity analysis. If reliable methods could be found to convert the HbA1C and GA to FPG, more published studies could be included in the current systematic review via the meta-analysis approach.

## Conclusions

A significant linear dose-response relationship between fasting plasma glucose concentration and the incidence risk of colorectal cancer was observed in our dose-response meta-analysis of prospective studies. Every 20 mg/dL increase in blood glucose concentration was associated with a 1.5% increase in the incidence of colorectal cancer. The role of blood glucose detection and control in preventing the rising incidence of colorectal cancer should be viewed critically considering that the statistically small effect and that the number of people with prediabetes or diabetes is large and still growing.

## Additional Information

**How to cite this article**: Shi, J. *et al.* A Linear Dose-Response Relationship between Fasting Plasma Glucose and Colorectal Cancer Risk: Systematic Review and Meta-analysis. *Sci. Rep.* **5**, 17591; doi: 10.1038/srep17591 (2015).

## Supplementary Material

Supplementary Information

## Figures and Tables

**Table 1:** The basic characteristics of studies included in this systematic review with meta-analysis.

Study	Region	Design	Cancer type	Gender (men%)	Baseline	Mean age (years)	Follow-up time (years)	Adjusted confounders
Schoen R. E., 1999*^ξ^	North America	Cohort	CRC	Both (42.4%)	1989–1990 (cohort1) 1992–1993 (cohort2)	73.9	6.4	Age, sex, and physical activity
Jee S. H., 2005**^ξ^**	Asia	Cohort	CRC	Male	1992–1995	45.3	10	Age, smoking, alcohol use
Jee S. H., 2005**^ξ^**	Asia	Cohort	CRC	Female	1992–1995	49.6	10	Age, smoking, alcohol use
Limburg P. J., 2006	Europe	Case-cohort	CC	Male	1985–1988	59	9	Age, smoking, alcohol use, BMI, diet, physical activity, history of DM
Limburg P. J., 2006	Europe	Case-cohort	RC	Male	1985–1988	59	9	Age, smoking, alcohol use, BMI, diet, physical activity, history of DM
Stocks T., 2011**ξ^¶^**	Europe	Cohort	CRC	Male	1972–2006	43.9	12.8	Age, smoking, BMI
Stocks T., 2011**ξ^¶^**	Europe	Cohort	CRC	Female	1972–2006	44.1	11.3	Age, smoking, BMI
Kabat G.C., 2012**^ξ^**	North America	Cohort	CRC	Female	1993–1998	64.3	11.9	Age, BMI, alcohol use, physical activity, family history of CRC, ethnicity
Wulaningsih W., 2012**^ξ^**	Europe	Cohort	CC	Both (57.7%)	1985–1996	43.84	8.5	Age, sex, SES, fasting status, glucose, total cholesterol
Wulaningsih W., 2012**^ξ^**	Europe	Cohort	RC	Both (57.7%)	1985–1996	43.84	8.53	Age, sex, SES, fasting status, glucose, total cholesterol
Nilsen T. L., 2001	Europe	Cohort	CRC	Male	1984–1986	48.5	10.8	Age
Nilsen T. L., 2001	Europe	Cohort	CRC	Male	1984–1986	49.8	10.8	Age
Ahmed R. L., 2006	North America	Cohort	CRC	Both	1987–1989	45–64	11.5	Age, sex, alcohol use, physical activity, family history of CRC, drugs used
Aleksandrova K., 2011 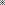	Europe	Nested case–control	CC	Male	1992–2000	58.8	3.7, 9.3	Age, follow-up time
Aleksandrova K., 2011 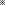	Europe	Nested case–control	RC	Female	1992–2000	58.1	3.7, 9.3	Age, follow-up time, menopausal status
Aleksandrova K., 2011 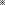	Europe	Nested case–control	CC	Male	1992–2000	58.8	3.7, 9.3	Age, follow-up time
Aleksandrova K., 2011 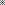	Europe	Nested case–control	RC	Female	1992–2000	58.1	3.7, 9.3	Age, follow-up time, menopausal status
Shin A., 2011	Asia	Cohort	RC	Male	1996–1997	30–80	7	Age
Shin A., 2011	Asia	Cohort	RC	Female	1996–1997	30–80	7	Age
Shin H. Y., 2014	Asia	Cohort	CRC	Male	2004-2011	42	4.7	Age, smoking, alcohol use, BMI, physical activity
Shin H. Y., 2014	Asia	Cohort	CRC	Female	2004-2011	41.5	4.7	Age, smoking, alcohol use, BMI, diet, physical activity

CRC = colorectal cancer, CC = colon cancer, RC = rectal cancer, BMI = body mass index, DM = diabetes mellitus, SES = socio-economic status.

*Cohort 1 (n = 5201; 5.3% members of minority groups) was recruited in 1989–1990; Cohort 2 with 687 minority subjects (97.8% African-American) was enrolled in 1992–1993.

**^ξ^**Site-specific (CC and RC) or sex-specific (male and female) analyses were not reported.

**^ψ^**The fasting state is defined according to the description of the original study.

**^¶^**Pooled analysis of 7 cohorts. Norwegian cohorts: The Oslo study I (Oslo), 1972–73, The Norwegian Counties Study, 1974–88, The Cohort of Norway, 1994–2003, The 40-year cohort (40-year), 1985–1999; The Austrian cohort: The Vorarlberg Health Monitoring and Prevention Programme, 1985–2005; and Swedish cohorts: The Vasterbotten Intervention Project, 1985 and ongoing, The Malmo Preventive Project, 1974–1992.

^※^The mean follow-up time was 3.7 years for cases and 9.3 years for controls. Follow-up time was adjusted for risk analysis.

**Table 2:** Study-specific and summary RRs for colorectal cancer, per 20 mg/dL increase in FPG.

Study			RR (95% CI)	*P* value
First author, year	Cancer type	Gender (men %)		
Schoen R. E., 1999	CRC	Both (42.4%)	1.036 (1.004–1.068)	0.025
Jee S. H., 2005	CRC	Male	1.016 (1.012–1.019)	0.000
Jee S. H., 2005	CRC	Female	1.008 (0.991–1.024)	0.351
Limburg P. J., 2006	CC	Male	1.094 (0.913–1.310)	0.332
Limburg P. J., 2006	RC	Male	1.102 (0.957–1.270)	0.177
Tanja Stocks, 2011	CRC	Male	1.023 (0.958–1.093)	0.492
Tanja Stocks, 2011	CRC	Female	1.054 (0.966–1.150)	0.240
GC Kabat, 2012	CRC	Female	1.092 (0.988–1.207)	0.085
Wulaningsih W., 2012	CC	Both (57.7%)	1.033 (1.006–1.061)	0.015
Wulaningsih W., 2012	RC	Both (57.7%)	0.964 (0.934–0.995)	0.022
Total	Total	Total	1.015 (1.012–1.019)	0.000

Notes: In the study-specific dose-response analysis, RRs were significant in 4 studies (*P* < 0.05) and not significant in 6 studies (*P* > 0.05). However, the total RR was significant without significant heterogeneity (*I*^*2*^ = 11%, *P* = 0.295). The *P* value was 0.303 for the test of linearity. Thus, the meta-analysis indicated that the incidence of colorectal cancer increased linearly with increasing FPG.

**Table 3:** Subgroup analyses of pooled relative risks of colorectal cancer per 20 mg/dL increase in fasting blood glucose.

Subgroup	Number of study	Relative Risk (95%CI)	*P* value	Test for heterogeneity*	
				*I*^*2*^ (%)	*P* value
Cancer type					
CRC	6	1.016 (1.012–1.019)	0.000	0	0.904
CC	2	1.035 (1.008–1.062)	0.011	11	0.295
RC**^ψ^**	2	1.031 (0.189–5.628)	0.972	11	0.345
Region**^ξ^**					
North America	2	1.041 (1.010–1.072)	0.008	2	0.381
Europe	6	1.010 (0.992–1.029)	0.284	16	0.26
Asia	2	1.015 (1.011–1.019)	0.000	0	0.694
Follow-up time (years)					
<10	5	1.015 (0.998–1.032)	0.076	46	0.030
≥10	5	1.015 (1.011–1.019)	0.000	0	0.928
Gender					
Both	3	1.013 (0.996–1.031)	0.122	3	0.405
Male	4	1.016 (1.012–1.020)	0.000	0	0.847
Female	3	1.011 (0.995–1.027)	0.164	0	0.555
Fasting status					
Fasting	6	1.016 (1.012–1.019)	0.000	0	0.585
Mix	4	1.008 (0.989–1.027)	0.421	23	0.208
Risk type^¶^					
HR	7	1.015 (1.011–1.019)	0.000	34	0.068
RR	3	1.036 (1.008–1.063)	0.010	0	0.941

^*^For the test of heterogeneity in each subgroup, we also calculated the *I*^*2*^ statistic, and 50% was regarded as the cutoff point for non-significant and significant levels. No significant heterogeneity was detected in our dose-response meta-analysis and we did not further test the heterogeneity between subgroups.

**^ψ^**The total sample size and the number of rectal cancer cases included were small.

^¶^Both studies using HR and RR as the risk type showed a significant dose-response relationship and we assigned RR as the risk type to illustrate our results.
