# Supplementary material for: A Linear Dose-Response Relationship between Fasting Plasma Glucose and Colorectal Cancer Risk: Systematic Review and Meta-analysis
Source: Sci Rep. 2015 Dec 1;5:17591. doi: 10.1038/srep17591 (PMC4665197; doi:10.1038/srep17591)
Supplement: Supplementary Information [file srep17591-s1.pdf]

A Linear Dose-Response Relationship between Fasting Plasma Glucose and Colorectal Cancer Risk: Systematic Review and Meta-analysis

Jianguo Shi[1], Lijuan Xiong[3], Jiaoyuan Li[2], Heng Cao[1], Wen Jiang[1], Bo Liu[1], Xueqin Chen[2], Cheng Liu[2], Ke Liu[1], Guobin Wang[1] and Kailin Cai[1]*

[1] Department of Gastrointestinal Surgery, Union Hospital, Tongji Medical College, Huazhong University of Science and Technology, Wuhan 430000, China.

[2] State Key Laboratory of Environment Health (Incubation), MOE (Ministry of Education) Key Laboratory of Environment &Health, Ministry of Environmental Protection Key Laboratory of Environment and Health (Wuhan), and Department of Epidemiology and Biostatistics, School of Public Health, Tongji Medical College, Huazhong University of Science and Technology, Wuhan 430000, China.

[3] Department of Infectious Disease, Union Hospital, Tongji Medical College, Huazhong University of Science and Technology, Wuhan 430000, China.

Detailed methods

Search strategy

Two investigators (Jianguo Shi and Lijuan Xiong) independently searched the PubMed and EMBASE databases before February 10, 2015 without language or date limitations. The basic retrieval scope that we used was sufficiently broad, and we include only human research (all participants with follow-up and subsequent colorectal cancer cases). The automatic species restriction was an important and reliable function for article classification in the two well-known medical databases. Thus, we used the automatic limiting function of the two databases to restrict our search to studies of humans. Additionally, we manually searched and screened the reference lists of pertinent articles and literature that cited eligible studies as of February 10, 2015.

Searching for PubMed

Search (((((((colorectal cancer) OR colorectal carcinoma) OR colorectal tumor) OR colorectal neoplasm) OR colorectal adenoma) OR colon cancer) OR rectal cancer) AND ((glucose) OR HbA1C) using the PubMed advanced search builder with no restriction (All Fields). And then we limited the above results to humans.

Here is the search details: ((((((("colorectal neoplasms"[MeSH Terms] OR ("colorectal"[All Fields] AND "neoplasms"[All Fields]) OR "colorectal neoplasms"[All Fields] OR ("colorectal"[All Fields] AND "cancer"[All Fields]) OR "colorectal cancer"[All

Fields]) OR ("colorectal neoplasms"[MeSH Terms] OR ("colorectal"[All Fields] AND "neoplasms"[All Fields]) OR "colorectal neoplasms"[All Fields] OR ("colorectal"[All Fields] AND "carcinoma"[All Fields]) OR "colorectal carcinoma"[All Fields])) OR ("colorectal tumour"[All Fields] OR "colorectal neoplasms"[MeSH Terms] OR ("colorectal"[All Fields] AND "neoplasms"[All Fields]) OR "colorectal neoplasms"[All Fields] OR ("colorectal"[All Fields] AND "tumor"[All Fields]) OR "colorectal tumor"[All Fields])) OR ("colorectal neoplasms"[MeSH Terms] OR ("colorectal"[All Fields] AND "neoplasms"[All Fields]) OR "colorectal neoplasms"[All Fields] OR ("colorectal"[All Fields] AND "neoplasm"[All Fields]) OR "colorectal neoplasm"[All Fields])) OR (colorectal[All Fields] AND ("adenoma"[MeSH Terms] OR "adenoma"[All Fields]))) OR ("colonic neoplasms"[MeSH Terms] OR ("colonic"[All Fields] AND "neoplasms"[All Fields]) OR "colonic neoplasms"[All Fields] OR ("colon"[All Fields] AND "cancer"[All Fields]) OR "colon cancer"[All Fields])) OR ("rectal neoplasms"[MeSH Terms] OR ("rectal"[All Fields] AND "neoplasms"[All Fields]) OR "rectal neoplasms"[All Fields] OR ("rectal"[All Fields] AND "cancer"[All Fields]) OR "rectal cancer"[All Fields])) AND (("glucose"[MeSH Terms] OR "glucose"[All Fields]) OR ("hemoglobin a, glycosylated"[MeSH Terms] OR "glycosylated hemoglobin a"[All Fields] OR "hba1c"[All Fields])) AND "humans"[MeSH Terms]

Searching for EMBASE

We searched the EMBASE database using the following methods with no language and time period limitation. There wasn't any restriction on each of the search item (all fields).

＃1 ((colorectal cancer or colorectal carcinoma or colorectal tumor or colorectal neoplasm or colorectal adenoma or colon cancer or rectal cancer) and (glucose.af. or HbA1C)).af.

＃2 limit 1 to human

Inclusion and exclusion criteria

Design

Prospective studies such as cohort, case-cohort or nested case-control studies were eligible for the meta-analysis. Retrospective and cross-sectional studies were excluded. We excluded intervention studies in which the intervention factors had a certain influence on colorectal cancer risk according to published studies. Otherwise, we further assessed them for eligibility on the basis of all of the inclusion and exclusion criteria.

Exposure and outcome

The exposure factor is blood glucose concentration with 3 or more categories in each eligible study for the dose-response meta-analysis. When one study had two blood glucose categories, we included it only in the highest compared to lowest meta-analysis. In our

meta-analysis, we excluded studies that mainly explored the association between treated diabetes and colorectal cancer risk, especially studies with only self-reported diabetes that did not test the blood glucose concentration of most participants. The blood glucose level of participants with diabetes mellitus was commonly influenced by hypoglycaemic agents and could not be considered greater than or equal to the diagnostic cutoff point for diabetes mellitus. All of the eligible studies reported the value of relative risks (RRs) or hazard ratios (HRs) and the corresponding numbers of participants and subsequent colorectal cancer cases for each of the blood glucose categories.

Participants

All of the participants needed to be at least 18 years old at the time of recruitment. Participants with a history of colorectal lesions were excluded at baseline. The numbers of participants and subsequent colorectal cancer cases needed to be available for each blood glucose category.

Data extraction and study quality assessment

Two investigators (Jianguo Shi and Lijuan Xiong) independently extracted the following data from eligible studies: first author, publication year, region of the recruited population, cancer type, study design, recruitment baseline, follow-up time, whether individuals with diabetes mellitus were excluded, age at recruitment, gender (men%), indicators to measure blood glucose levels and related doses (mainly fasting plasma glucose (FPG) and HbA1C), fasting state, the size of the observational population (N) and the number of colorectal cancer cases, the types and values of relative risk and 95% confidence intervals, and adjusted confounders. For the cancer type and gender items, we divided one original study into two different studies if it contained full site-specific (colon cancer and rectal cancer) and sex-specific (male and female) data. In these cases, separate data, instead of data for colorectal cancer or for both sexes, were extracted from those studies. If an article reported results for different genders, we also considered them separately. In terms of FPG, fasting is currently defined as having no caloric intake for at least 8 h. However, we recorded the fasting state in light of the definition in original studies considering the time and regional differences among eligible studies. If several models, such as crude models and adjusted models, were reported in the included articles, we analysed the model with adjustment for many of the confounders. We evaluated the quality of each study with the Newcastle-Ottawa Quality Assessment Scale for cohort studies[1] (see Figure 1 below).

Data synthesis and analysis

The mean blood glucose concentration of each category was assigned as the category exposure dose. When we reported only a range of blood glucose in each category, we adopted the midpoint between cutoff points as the category exposure dose. When the lower bound of the lowest category was not delimited, we calculated the average value of the upper bound

and 70 mg/dL (3.9 mmol/L) for fasting plasma glucose (FPG) or 4% for haemoglobin A1C (HbA1C), which was the lower limit of normal blood glucose concentration[2,3]. When the upper bound of the highest category was unknown, the category exposure dose was assigned as the lower bound plus 1.5 times the width of the preceding interval. We used unified FPG as the exposure factor because it is a common diagnostic criterion for prediabetes and T2DM mellitus and is a common laboratory value for describing metabolic syndrome. Furthermore, most of the prospective studies included in our study reported FPG as the exposure factor. None of the studies used only the 2-h plasma glucose (2-h PG) value after a 75 g oral glucose tolerance test (OGTT) as the blood glucose exposure. We attempted to convert HbA1C into FPG via the following model: the FPG and HbA1C lower cutoff points for prediabetes were 100 mg/dL and 5.7%, respectively; for T2DM, they were 126 mg/dL and 6.5%[4]. Thus, we assumed that 4%, 5.7%, and 6.5% HbA1C were equal to 70 mg/dL, 100 mg/dL, and 126 mg/dL FPG, respectively. These approaches were used in another dose-response meta-analysis[5]. All of the studies that reported only HbA1C as the blood glucose concentration were discarded because there were no 4%, 5.7%, or 6.5% doses in the extracted data for any of these studies[6-9]. One study assigned glycoalbumin (GA)[10] as the exposure state of blood glucose concentration. Furthermore, we could not obtain sufficient scientific evidence to convert GA into FPG. Therefore, the outcome for this meta-analysis was the association between the incidence risk for colorectal cancer and fasting plasma glucose exposure.

We used the generalized least squares trend (GLST) model proposed by Greenland and Longnecker[11,12] to estimate the trend in the effect. Based on the construction of an approximate variance-covariance matrix for log relative risks, this approach can isolate a corrected linear association using general least squares. A cubic spline model with 3 knots at the 25%, 50% and 75% percentiles of the distribution was established to explore the potential non-linear relationship between glucose concentration and colorectal cancer, and a P value for non-linearity was calculated by testing the null hypothesis that the coefficient of the second spline was equal to zero[13].

The heterogeneity among studies was assessed using Cochran's Q test and the I^2 statistic. The criterion for identifying heterogeneity was a P value less than 0.05 for the Q test or an I^2 value greater than 50%. When significant heterogeneity was detected, data from the included studies were combined via a random-effects model; otherwise, a fixed-effects model was employed. We conducted subgroup analysis to search for the source of heterogeneity, and the subgroups were pre-specified mainly according to cancer type, gender, region and follow-up time. Sensitivity analysis was also conducted to evaluate the stability of the association. Moreover, we conducted a meta-analysis of studies with two categorical variables (highest compared to lowest blood glucose). Publication bias was examined via Begg's and Egger's regression tests.

All of the analyses were performed using Stata 10.0 software. All of the P values were two-sided, and $P < 0.05$ was considered statistically significant.

Supplementary Results

Justifications for exclusion

Although the issue of whether colorectal cancer influences the prevalence of diabetes mellitus is uncertain[14,15], we excluded retrospective and cross-sectional studies in case of potential reverse causality. Therefore, we first excluded 2,761 articles by screening the titles and abstracts of studies that were non-prospective studies or studies whose research outcomes were not related to the incidence risk of colorectal cancer. Next, reviews and meta-analyses, statements, letters and editorials (n=14) were excluded, and 3 articles were added after a detailed evaluation of full-text articles. No new eligible references were found in the 17 articles. Non-prospective studies (n=7) were further excluded because we could not confirm the detailed research methods only by screening the titles and abstracts. Moreover, 24 articles were eliminated because there were no specific exposure doses of blood glucose, because we could not obtain data on the number of colorectal cancer cases for each exposure dose, or because there were no data on the RR or HR for each blood glucose category. For 4 studies, the number of cases/total cohort or adjusted confounders was smaller than the studies from the same cohort. The 428 citing articles that were manually added were excluded because they were duplicated with the initial search or because they met the above criteria for exclusion. Five articles were excluded because the dose-response meta-analysis required at least three exposure categories[16]. Although there were some studies on the conversion between HbA1C or GA and FPG[17,18], we still eliminated the HbA1C or GA studies because we could not accurately perform the conversion considering the characteristics of our extracted data.

Figure 1 | Quality assessment of each eligible study according to the Newcastle-Ottawa Quality Assessment Scale for cohorts study

Study	Selection				Comparability	Outcome		
	1	2	3	4	1	1	2	3
Schoen R. E., 1999*		★	★	★	★★	★	★	★
Jee S. H., 2005	★	★	★	★	★★	★	★	★
Limburg P. J., 2006*		★	★	★	★★	★	★	★
Stocks T., 2011	★	★	★	★	★★	★	★	★
Kabat G.C., 2012*		★	★	★	★★	★	★	★
Wulaningsih W., 2012	★	★	★	★	★★	★	★	★
Nilsen T. L., 2001	★	★	★	★	★★	★	★	★
Ahmed R. L., 2006	★	★	★	★	★★	★	★	★
Aleksandrova K., 2011	★	★	★	★	★★	★		★
Shin A., 2011	★	★	★	★	★★	★	★	★
Shin H. Y., 2014	★	★	★	★	★★	★		★

For selection: 1. Representativeness of the exposed cohort (★ somewhat representative of the average level in the recruitment region). * The average age of participants in these studies was older at recruitment time. 2. Selection of the non-exposed cohort (★ all the non-exposed cohorts were drawn from the same communities as the exposed cohorts). 3. Ascertainment of exposure (★ the blood glucose concentration was acquired from laboratory report). 4. Demonstration that outcome of interest was not present at start of study (★ yes).
For comparability: 1. Comparability of cohorts on the basis of the design or analysis (★ the most important factor was adjusted for analysis; ★★ a second important factor was adjusted for site-specific or sex-specific studies. We assigned age and gender as the most important and second important confounders).
For outcome: 1. Assessment of outcome (★ the diagnosis of most colorectal cancer cases came from medical records). 2. Follow-up long enough for outcomes to occur (★ the average value of follow-up time > 5 years for eligible studies). 3. Adequacy of follow up of cohorts (★ the mean proportion of subjects lost to follow up was less small).

Table 1 | Incidence risks for colorectal cancer of various fasting plasma glucose doses in each study

Fasting plasma glucose (mg/dl)	Cancer type	Gender (male%)	Excluded DM	NO. of cases / total	Relative Risk	
					type	values (95% CI)
Schoen R. E., 1999	CRC	Both (42.4%)	no		RR	
75.3				22/1591		1.00
98				18/1352		0.9 (0.50-1.80)
106.9				29/1433		1.4 (0.80-2.40)
340.75				32/1393		1.8 (1.00-3.10)
Jee S. H., 2005	CRC	Male	no		HR	
<90				6797/429370		1.00
90-109				6837/304362		1.08 (1.01-1.15)
110-125				7674/58020		1.14 (1.02-1.27)
126-139				7928/11459		1.03 (0.82-1.28)
≥140				8522/26559		1.13 (0.98-1.30)
Jee S. H., 2005	CRC	Female	no		HR	
<90				3290/270157		1.00
90-109				3310/157940		1.04 (0.94-1.15)
110-125				3282/22578		1.11 (0.92-1.34)
126-139				2991/5657		0.80 (0.53-1.20)
≥140				3201/12283		1.07 (0.84-1.36)
Limburg P. J., 2006	CC	Male	no		HR	
<92				8/107		1.00
93-98				14/114		1.67 (0.59–4.73)
99-107				15/116		1.92 (0.66–5.58)
>107				10/109		1.38 (0.42–4.52)

Limburg P. J., 2006	RC	Male	no		HR
<92				15/114	1.00
93-98				18/118	1.03 (0.42–2.51)
99-107				29/130	2.09 (0.89–4.91)
>107				25/124	1.85 (0.77–4.45)
Stocks T., 2011$	CRC	Male	no		RR
75.6				490/54782	1.00
86.4				503/56236	1.00 (0.63-1.55)
91.8				537/50451	1.19 (0.77-1.86)
99				630/63455	1.11 (0.74-1.70)
124.2				668/64942	1.15 (0.74-1.75)
Stocks T., 2011$	CRC	Female	no		RR
73.8				283/51127	1.00
82.8				293/49470	1.07 (0.58-1.92)
90				392/63800	1.11 (0.63-1.92)
95.4				368/57811	1.15 (0.66-2.03)
117				520/66626	1.41 (0.83-2.35)
Kabat G.C., 2012¶	CRC	Female	yes		HR
<89.5				18/1538	1.00
89.5-99.5				28/1669	1.32 (0.72-2.40)
≥99.5				35/1695	1.74 (0.97-3.15)
Wulaningsih W., 2012$	CC	Both (53.73%)	no		HR
<79.2				310/77290	1.00
79.2-86.4				572/125097	1.14 (1.00-1.32)
86.4-93.6				622/138464	1.12 (0.98-1.29)
≥93.6				968/199458	1.21 (1.05-1.40)
Wulaningsih W., 2012$	RC	Both (53.73%)	no		HR
<79.2				242/74604	1.00
79.2-86.4				338/122589	0.85 (0.72-1.00)
86.4-93.6				346/138527	0.77 (0.65-0.91)
≥93.6				584/204589	0.88 (0.74-1.05)
Nilsen T. L., 2001*	CRC	Male	no		RR
<144				321/182295P	1.00
≥144				21/11302P	0.98 (0.58-1.40)
Nilsen T. L., 2001*	CRC	Female	no		RR
<144				316/208261P	1.00
≥144				25/6502P	1.98 (1.31-2.98)
Ahmed R. L., 2006*	CRC	Both (44.6%)	no		RR
<100				79/86190P	1.00
≥100				115/78100P	1.39 (1.00-1.80)
Aleksandrova K., 2011	CC	Male	no		RR
<100				134/307	1.00
≥100				178/317	1.85 (1.28-2.66)
Aleksandrova K., 2011	RC	Male	no		RR

<100				103/220	1.00
≥100				116/218	1.66 (1.00-2.73)
Aleksandrova K., 2011	CC	Female	no		RR
<100				199/451	1.00
≥100				178/303	2.80 (1.86-4.22)
Aleksandrova K., 2011	RC	Female	no		RR
<100				96/222	1.00
≥100				89/148	3.20 (1.78-5.77)
Shin A., 2011	RC	Male	no		HR
<126				1376/816145	1.00
≥126				159/53351	1.30 (1.10-1.50)
Shin A., 2011	RC	Female	no		HR
<126				506/375690	1.00
≥126				45/19730	1.20 (0.90-1.60)
Shin H. Y., 2014*	CRC	Male	yes		HR
<100				145/375362.6P	1.00
≥100				82/100275.2P	1.51 (1.11-2.05)
Shin H. Y., 2014*	CRC	Male	yes		HR
<100				76/302313P	1.00
≥100				17/38191.9	1.23 (0.67-2.26)

RR = Relative risk, HR = Hazard ratio

$The number of observational population in each blood glucose category was calculated using the relative risk and the corresponding number of cases and the total number of participants.

¶The study excluded women who at baseline had a diagnosis of diabetes, who were taking diabetes medication and who had a fasting blood glucose ≥126 mg/dL.

*These studies reported the data on person year of exposure. P = Person year

According to the values of RR or HR for each category in every eligible study, the associations between fasting plasma glucose and incidence risk of colorectal cancer were varied among these individual studies.

Table 2 | Dose-response meta-analysis on relative risks for colorectal cancer incidence per 20 mg/dL increase in FPG, each study removed in turn. §

Study removed			Pooled RR (95% CI) of remaining studies	*P* value	Test for heterogeneity	
First author, year	Cancer type	Gender (men %)			I^2 (%)	*P* value
Schoen R. E., 1999	CRC	Both (42.4%)	1.015 (1.011-1.019)	0.000	13	0.268
Jee S. H., 2005	CRC	Male	1.013 (1.002-1.025)	0.021	16	0.227
Jee S. H., 2005	CRC	Female	1.016 (1.012-1.020)	0.000	13	0.267
Limburg P. J., 2006	CC	Male	1.015 (1.012-1.019)	0.000	16	0.224

Limburg P. J., 2006	RC	Male	1.015 (1.012-1.019)	0.000	8	0.340
Stocks T., 2011	CRC	Male	1.015 (1.012-1.019)	0.000	21	0.161
Stocks T., 2011	CRC	Female	1.015 (1.012-1.019)	0.000	19	0.188
Kabat G.C., 2012	CRC	Female	1.015 (1.011-1.019)	0.000	10	0.310
Wulaningsih W., 2012	CC	Both (57.7 %)	1.015 (1.011-1.019)	0.000	13	0.271
Wulaningsih W., 2012	RC	Both (57.7 %)	1.016 (1.012-1.020)	0.000	0	0.878

§ The tests of linear trend were still significant after omitting each study in turn and the *P* values varied from 0.112 to 0.835 (not showed in the table 2).

Another sensitivity analysis was conducted by excluding the exposure categories including fasting blood glucose ≥ 126mg/dL (the FPG diagnostic cut point for diabetes mellitus) from all the 10 studies. There was also a linear dose-response relation ($P = 0.092$) between FPG and incidence risk of colorectal cancer and the summarized RR was 1.015 (95% CI: 1.011-1.019, $P = 0.000$) with no significant heterogeneity ($I^2 = 19$, $P = 0.213$).

References

1 Wells, G. A. et al. The Newcastle-Ottawa Scale (NOS) for assessing the quality of nonrandomised studies in meta-analyses.(2011). Available at: www.ohri.ca/programs/clinical_epidemiology/oxford.asp. Accessed 26/03/ 2015.

2 Cryer P. E. & Davis S. N. Hypoglycemia in *Harrison's Principles of Internal Medicine 18th edn* (eds Longo D. L. *et al.*) Ch. **345**, (McGraw-Hill, 2012).

3 Baumann G. P. & Jameson J. L. Laboratory Values in *Harrison's manual of medicine 18th edn* (eds Longo D. L. *et al.*) Ch. **220**, 1411 (McGraw-Hill, 2013).

4 Association, A. D. Diagnosis and classification of diabetes mellitus. *Diabetes Care* **37**, S81-90, (2014).

5 Liao, W. C. *et al.* Blood glucose concentration and risk of pancreatic cancer: systematic review and dose-response meta-analysis. *Brit Med J* **349**, g7371 (2015).

6 Platz, E. A. *et al.* Glycosylated hemoglobin and risk of colorectal cancer and adenoma (United States). *Cancer Cause Control* **10**, 379-386 (1999).

7 Saydah, S. H. *et al.* Association of markers of insulin and glucose control with subsequent colorectal cancer risk. *Cancer Epidem Biomar* **12**, 412-418 (2003).

8 Stocks, T. *et al.* Components of the metabolic syndrome and colorectal cancer risk; a prospective study. *Int J Obesity* **32**, 304-314 (2008).

9 Rinaldi, S. *et al.* Glycosylated hemoglobin and risk of colorectal cancer in men and women, the European prospective investigation into cancer and nutrition. *Cancer Epidem Biomar* **17**, 3108-3115 (2008).

10 Ozasa, K. *et al.* Glucose intolerance and colorectal cancer risk in a nested case-control study among Japanese people. *J Epidemiol* **15**, S180-S184 (2005).

11 Greenland, S. & Longnecker, M. P. Methods for trend estimation from summarized dose-response data, with applications to meta-analysis. Am J Epidemiol **135**, 1301-1309 (1992).

12 Orsini, N., Bellocco, R. & Greenland, S. Generalized least squares for trend estimation of summarized dose–response data. Stata J **6**, 40-57 (2006).

13 Smith, P. L. Splines as a Useful and Convenient Statistical Tool. Am Stat **33**, 57–62 (1979).

14 Aggarwal, G., Kamada, P. & Chari, S. T. Prevalence of diabetes mellitus in pancreatic cancer compared to common cancers. Pancreas **42**, 198-201 (2013).

15 Ahmadi, A., Mobasheri, M., Hashemi-Nazari, S. S., Baradaran, A. & Choobini, Z. M. Prevalence of hypertension and type 2 diabetes mellitus in patients with colorectal cancer and their median survival time: A cohort study. J Res Med Sci **19**, 850-854 (2014).

16 Orsini, N., Li, R., Wolk, A., Khudyakov, P. & Spiegelman, D. Meta-analysis for linear and nonlinear dose-response relations: examples, an evaluation of approximations, and software. Am J Epidemiol **175**, 66-73 (2012).

17 Kumaravel, B. et al. Use of haemoglobin A1c to detect impaired fasting glucose or Type 2 diabetes in a United Kingdom community based population. Diabetes Res Clin Pract **96**, 211-216 (2012).

18 Jung, C. H. et al. Development of an HbA1c-based conversion equation for estimating glycated albumin in a Korean population with a wide range of glucose intolerance. Plos One **9**, e95729 (2014).
